# Tourism destinations and tourist behavior based on community interaction models of film-enabled tourism destinations

**DOI:** 10.3389/fpsyg.2022.1108812

**Published:** 2023-02-07

**Authors:** Yongshi Lao, Jianfei Zhu, Jinlin Liu

**Affiliations:** ^1^School of Management, Shenzhen University, Shenzhen, China; ^2^School of Management Science and Engineering Administration, Guizhou University of Finance and Economics, Guiyang, China

**Keywords:** cultural tourism, interest-related community, film-enabling, tourism experience, grounded theory

## Abstract

The importance of media-induced tourism has increased in the latest decade. The integration and collaboration of film elements is an especially effective pathway for the innovative development and upgrading of the experience of the cultural tourism industry. Existing studies on the mechanism of tourism destinations and cultural tourism development, mainly from the perspective of tourism destinations and tourist experience, have rarely explored the cultural tourism development mechanism from the perspective of interest-related community interaction in the film-enabling context. In this study, we conducted high-frequency word analysis and element category analysis of the online text data of the Japanese animation film *Your Name* from the angle of interest-related community interaction through utilizing online text analysis and Grounded Theory analysis. Based on the interest-related community interaction, we analyzed the elements of interest-related community interaction in cultural tourism triggered by the film, including tourist hotspots, tourism resources, the tourist experience, sightseeing expectations, tourism evaluation and information dissemination, and formulated the orientation pathway of film-enabling cultural tourism. In this study, we aimed to enrich cultural tourism research and provide a reference point and theoretical support for film-enabling cultural tourism in the Internet era by introducing the concept of interest-related community innovation to the scene of film-enabling cultural tourism.

## 1. Introduction

With the vigorous development of cultural tourism, the combination of cultural factors with tourism destinations, such as sports, music and film with tourism destinations, has become an important development in the current cultural tourism industry (Ferreira et al., 2022; Hwang et al., 2022; Jin et al., 2022). Film can induce tourism behaviors, thus the “film + cultural tourism” model is also favored by both destination hosts and tourists: for example, Japan, Thailand, and the U.S. have become popular film-enabled tourism destinations (e.g., Busby et al., 2013, p. 571; Wen et al., 2018, p. 218; Du et al., 2020, p. 367). The innovation of Internet technology has broken the limitation of communities in time and space, and consequently, more and more film lovers gather due to their similar interests and hobbies, form a community of shared film interests, and become inheritors and promoters of film culture. Interest-related community is a virtual social relationship based on shared interest. It is a community with both traditional community cohesion and modern vitality.

With mature techniques, vivid stories, beautiful human settings and industry-leading picture quality, the Japanese animation film *Your Name* released in 2016 won a warm response from Chinese consumers. With the continuous upsurge of film popularity, the tourism industry derived from it developed rapidly. After the original filming location of *Your Name*, Nagano in Japan, was successfully selected as one of the top 10 best tourism destinations by the United States news channel CNN, a vast number of film lovers became fascinated with the place, and the cohesion of the interest-related community was also improved greatly. According to Japanese media reports, Hida City of Gifu-ken in Japan, one of the shooting places, despite its remote location, witnessed an influx of Chinese tourists after the film was released. The number of foreign tourists doubled, and about 80% of them came for the holy land tour mentioned in *Your Name*. As the data provided by the Hida Tourism Association indicated, the number of tourists in Hida City exceeded 1 million in 2016 (April 2016–March 2017) and reached 1.13 million in 2017, creating an economic benefit of 18.5 billion yen for Gifu-ken where Hida City is located in a short time. There were 6,984 foreigners out of the 1 million tourists in 2016 who stayed for accommodation, up 27.3% year on year. Among them, Hong Kong, China’s Mainland and Taiwan were the top three regions where the tourists came from. Data released by the Japan National Tourism Organization showed that a total of 30 million foreign tourists visited Japan in 2018, 14 percent of whom went to Japan because of the attraction of the anime (Chen et al., 2021, p. 1040).

The research into interest-related community is of great academic and practical value to tourism destination management and marketing (Taecharungroj, 2022; Wong et al., 2022). Therefore, it has become a focus of the film and television industry to study the enabling mechanism of film and television cultural tourism based on the rules of interest-related community interaction, and it has also become an important topic discussed by academia. The mechanism of promoting cultural tourism development through the community interaction of shared film interest also triggered deep thinking on the “film and television + cultural tourism” model (Du et al., 2020; Marques de Sousa et al., 2022). Over the last decades, various incentives have emerged in cultural tourism research, especially in cultural tourism motivation research and tourist destination marketing research (Cuccia and Rizzo, 2011 p. 590; Connell, 2012, p. 1009; Coshall et al., 2015, p.1617; Boyd, 2022, p. 220). In terms of tourist destination marketing, emotional appeal is the most popular incentive, and it is a major strategy to “tell stories.” Story marketing encourages audiences to form a positive image of the tourist destination in their minds through inspiring stories and plots in the film, and the audiences are guided to learn more about and to visit the tourist destination (Li and Liu, 2020, pp. 382–383; Li et al., 2021, p. 1810). In addition, researchers have recognized the role of tourist participation the tourism experience and behavior (Kim et al., 2017, p. 869; Chen and Rahman, 2018, p. 10; Chen, 2021, p. 1911). Most studies on film culture tourism explore the tourists’ perception and behavior of film tourism through the participation of tourists (Kim et al., 2019b, p. 285) or celebrity attachment (Tengand Chen, 2020; Thelen et al., 2020, p. 292). Some scholars have divided the impacts of tourist behaviors on film tourism into two stages, namely, the “production period” (the direct impact of filming activities during film production) and “post-production” (the film tourism activities and experiences generated by the influx of film tourists after film production and the subsequent public relations of film tourism). Existing studies have shown that the authenticity of films and celebrity attachment plays a crucial role in the process of tourism activities triggered by films (Wong and Lai, 2015, p. 163; Liu et al., 2021, p. 2941).

At present, the vast majority of studies on film tourism focus on Western cases, and few involve Asian film tourism cases (Rittichainuwat et al., 2018, p. 1275). With the innovative development of global films, film production is no longer dominated by Western media, and Asian culture is also more widely disseminated through films. Although there are a great number of studies on film and television cultural tourism, few of them have been conducted from the perspective of interest-related community interaction (Kirillova et al., 2019; p. 270; Rather et al., 2019, p. 520). With the development of Internet technology, Internet users have transformed from passive readers to active content creators. They have built an online virtual tourism community, and their online communication has generated the so-called user-generated content (UGC), and the interest-related community has shaped an open and shared tourism experience (Du et al., 2020, p. 367).

Given the above consideration, the current study applied online text analysis and element coding of Grounded Theory to conduct high-frequency word analysis and element category analysis toward the text data of the Japanese animation film *Your Name*. The ROST-CM6 software was used to crawl the online text review data in China. In view of the keyword characteristics of the text data, the acquired data were divided into perspectives of the tourist destination hosts and the tourists, and the top 30 high-frequency words in terms of interactive discourse frequency were conceptualized to form a core generic category, and their generic analysis was conducted through three-tier coding (open coding, relational coding, and core coding). Furthermore, in line with the characteristics of tourism activities between China and Japan, combining the film-enabling tourism characteristics from the perspective of the tourist destination hosts and the tourists, this study proposed the elements of the interest-related community interaction of cultural tourism triggered by the film *Your Name*, namely, tourist hot spots, tourism resources, tourist experiences, sightseeing expectations, tourism evaluation and information dissemination, and finally a film tourism enabling pathway was generated based on the interest-related community interaction, thus providing theoretical support and reference for the exploration of film-enabling tourism mechanism in the Internet era.

## 2. Literature review and research hypotheses

### 2.1. Cultural tourism

Cultural tourism can be defined as “the movement of persons to cultural attractions away from their normal place of residence, with the intention to gather new information and experiences to satisfy their cultural needs” (Chen and Rahman, 2018 pp. 153–163; Li and Katsumata, 2020, p. 20; Pérez García et al., 2021, p. 315; Yang et al., 2022) and involves “tourists experiencing and having contact with a host population and its cultural expressions, experiencing the uniqueness of culture, heritage and the characters of its place and people” (Lynch et al., 2011, p. 979). This definition implies that cultural tourism allows tourists to engage, explore, and enjoy the difference of tourism destinations and the way they compare to their own home identities, encouraging self discovery (Mueller et al., 2009, p. 65).

The literature has emphasized that earlier cultural tourism emerged from the development and operation of cultural resources, such as heritage tourism, religious tourism, and folklore tourism (Cisneros-Martinez and Fernandez-Morales, 2015, p. 776; Figini and Vici, 2012, p. 837; Ruiz-Ballesteros and Hernández-Ramírez, 2007, p. 680) Socio-cultural impacts of tourism result from the interactions between people. Tourists interact with service staff, tour leaders, other tourists, and local residents (Minkiewicz et al., 2014, p. 33; Weiler and Black, 2015, p. 367). Additionally, the interactions are not limited to people. Tourists also interact with objects in environments based on experiences, such as sports, heritage sites, music, and even specific atmospheres (Li and Katsumata, 2020, p. 20; Ferreira et al., 2022, p. 337; Minkiewicz et al., 2014, p. 30–59; Prebensen and Foss, 2011, pp. 11). Thanks to changes in technology, increasing productivity has made it possible for more cultural resources to enter the tourism market, such as theme parks and film culture (Connell, 2012, p. 1009; Hu et al., 2021). Therefore, cultural tourism has become a major market in the tourism industry.

Cultural tourism has been a core element of the tourism industry for destination marketing and promotion (Du et al., 2020; Li and Katsumata, 2020). However, most studies focus on either residents’ views or tourists’ interest in cultural tourism. Few studies have focused on the interaction between residents and tourists in the context of film-enabled tourism. Second, cultural tourism is mostly represented by visiting historical sites and museums, and there is a lack of research that combines it with new cultural communication channels such as movies and music. Månsson highlighted the importance of the convergence of a range of media products in propelling tourism (Marques de Sousa et al., 2022, p. 526). Therefore, the introduction of film elements into research is conducive to promoting the development of tourism.

### 2.2. Film tourism

The term “film tourism” originated from the United Kingdom and the United States in the 1990s, originally referring to a new type of tourism formed by combining film and television bases with characteristic tourism (Riley, 1998, p. 922). It caters to the needs of contemporary tourists who pursue novelty and desire to combine knowledge, appreciation and dream-seeking psychology. Meanwhile, it integrates organically film elements into the tourism industry to form tourism products that can be seen, felt, and experienced (Kim, 2018, p. 262).

In the literature on film-induced cultural tourism, extant research has been dominated by locational shoots in either the U.S. or UK. The early highlight of the western market is partly explained by the dominance of Hollywood in filmmaking (Connell, 2012, pp. 1007–1,009). In the past two decades, empirical evidence reports that film production is no longer dominated by the US media but is increasingly shifting toward Asian market (Nanjangud, 2019, p. 5; Lin et al., 2021; Nakayama, 2021, p. 70; Wang et al., 2021, pp. 717–719). Academia and industry show increasing interest in Asian culture, and the development and transmission through film is encouraging the wider transmission of geography and socio-cultural values, all of which create significant implications for the tourism sector. One of the earliest papers to explore the potentially lucrative effects of film tourism for a destination was that by Cohen (1986), who recognized the power of film in motivating tourist demand. Similarly, research also argued that the influence of film and television on tourism destinations would increase (Du et al., 2020, p. 367).

Film tourism activities include reenacting scenes, tasting the food and beverages that appeared in the film, and experiencing the culture represented therein (Kim, 2012, p. 389). This type of experience is unique (Terzidou et al., 2018, p. 309). Connell outlined a range of product and service innovations in the retail, catering and accommodation sectors (Connell, 2012, pp. 1007–1,009). Examples of themed innovation of products include hotel packages with inclusive film tours, production of souvenirs, stage set or studio tours, themed food and drink, and photography opportunities at key film sites. However, at present, the combination of film and tourism is no longer limited to product innovation. The Internet has eliminated the boundary between them. Individuals have formed an interest-related community in the network society and built a new virtual relationship network. The barriers associated with film culture communication and tourism activities have been broken. Film elements based on the interaction of interests have offered tourism activities an innovative format. Scholars have pointed out that it may not simply be films that create tourist interest but the consumption of a number of media products and consumer-to-consumer interactions. Therefore, research into the film-enabling tourism mechanism of interest-related community interaction has become a new topic of great significance.

### 2.3. Interest-related community

The idea of community is predicated on a collective sense of common interests and purpose. Members of a community are bound together through comradeship and a desire to seek one another out. In the past, communities were based, at least in their conception, on proximity: members lived near one another (Lysloff, 2003, pp. 233–236). Fischer further elaborated on the role of social networks in residents’ life, pointing out that residents living in non-adjacent areas form a group through specific relationships (such as common interests or hobbies, common values, etc.), thus forming their own social networks (Fischer, 1977, pp. 764–767). A community coheres through the common interests, ideals, and goals of its membership.

Furthermore, virtual communities are defined as aggregations of Internet users who form webs of personal relationships. Virtual communities have their own cultures and expectations (Kannan et al., 2000, pp. 415–416). The community stays together by adhering to the values and norms of the group (Spaulding, 2010, pp. 38–39). The Internet lays the groundwork for community by providing access to sustained communication, informational resources, and most importantly, a common locus for members to gather. Internet communities have emerged, despite temporal and spatial displacements, because they are formed entirely out of social relationships that are very real to members: relationships emerging out of communication, exchange, common interests and purpose, and mutual commitment. Boisvert and Milette (2009) proposed from the perspective of “Collective Intelligence” that members may shift from one community to another as their interests and needs change and they may belong to more than one community at the same time. Yet, they are held together through the mutual production and reciprocal exchange of knowledge (Boisvert and Milette, 2009, pp. 183–185). Scholars also noticed the social exchange phenomenon of interest groups in online interactions. Baker pointed out through participatory observation of rock fans in the virtual community that the exchange of objects may solidify bonds within the group (Baker, 2012, pp. 519–521). These objects that are bought, traded, and gifted will likely reflect the values and norms of the particular culture. And the resulting transactions serve to strengthen the community purposes of sharing information and resources and to connect the members, sometimes in intimate friendships that develop to pursue and enjoy activities.

### 2.4. Research hypotheses development

Given the above review, the current domestic research into online communities is still limited to concept analysis, and few studies have explored the development, interaction and maintenance of social relations among members of virtual online communities in depth. Meanwhile, film tourism is presenting a new format in the “Internet plus” era, but there is a lack of studies on the integration and development of film elements and tourism activities in the new media environment. Based on the Tourism Gaze Theory, taking the Japanese animation film *Your Name* as an example, this study explored the characteristics of the tourist destination hosts and tourists through the discourse construction analysis of the film interest-related community and discussed the interactive characteristics of the interest-related community in the process of film-enabling tourism in combination with travelling forms in order to make an in-depth exploration of the internal mechanism of film-enabling tourism. Specifically, the current study focused on the following issues:

Q1: Analysis of film-enabling tourism characteristics from the perspective of the tourist destination hosts;

Q2: Analysis of film-enabling tourism characteristics from the perspective of tourists;

Q3: Exploration of the pathway mechanism of film-enabling tourism.

## 3. Research methodology and sample selection

### 3.1. Research context: Your name

*Your Name* is a youth film directed by the famous Japanese animation director Makoto Shinkai, demonstrating the classic theme of emotional development of the characters in the context of changes in time and space. Reflecting the strong filming style of Makoto Shinkai, it is rich in animated scenes that reflect real life and real-life filming locations. It has sparked a large-scale cultural tourism trend, and it is a key case for cultural tourism research. *Your Name* tells a story of exchanging souls through time and space. Miyamizu Mitsuha, a young girl living in the countryside of Japan, and Tachibana Taki, a young boy living in Tokyo, exchange souls through dreams, experience each other’s lives, and generate subtle feelings for each other. The whole setting integrates multiple dimensions such as dreams, time, and space, and this is expanded on in the plot twists and the depth of thought of the film, and this contributes to the popularity of the shooting sites as tourism destinations.

The analysis of discourse characteristics is an effective way to explore the interaction rules of interest-related community. The current study mainly applied the ROST-CM6 software to conduct online text analysis, excavated the whole network data of the Japanese animation film *Your Name*, and thus reproduced and built the discourse structure of the interest-related community. Besides, Grounded Theory was adopted to analyze the film-enabling tourism characteristics from the perspective of the tourist destination hosts and the tourists and to further explore the overall characteristics of the film-enabling tourism mechanism. The data samples from the whole network can effectively reflect the characteristics of topics about film and tourism, map the characteristics of the interest-related community, and reflect the characteristics of the film-enabling tourism mechanism. This study therefore randomly collected 20,567 pieces of information from 2 December 2016 to 2 August 2020. These samples were mainly from platforms like Weibo, WeChat, Today’s Headlines, portal websites, etc. During this period, the development trend of discourse was gentle and stable, and this could reflect the issues discussed in this study. After completing data collection, the authors cleaned and sorted out the data by manually eliminating the wrong data, incomplete data, advertisements and other text materials irrelevant to the research to ensure the accuracy and reliability of the research results, and finally 20,074 entries of valid text data were obtained.

### 3.2. Research framework

**Figure 1:**
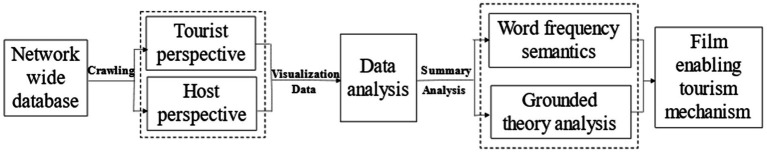
Technical route.

The technical route of problem exploration in this study is shown in Figure 1. First of all, the ROST-CM6 software was used to crawl the whole network data to lay the analysis foundation for the research; Second, in view of the crawled keyword characteristics, the acquired data were divided into two groups from the perspective of the tourist destination hosts and the perspective of tourists, and the two groups were summarized and analyzed in line with Grounded Theory; Finally, on account of the characteristics of Chinese and Japanese tourism activities, and in combination with the film-enabling tourism characteristics from the perspective of the tourist destination hosts and the perspective of tourists, the film-enabling tourism mechanism was explored, and research conclusions and suggestions were put forward.

## 4. Data analysis and research results

### 4.1. High-frequency words analysis

In line with textual attribute characteristics, 20,074 entries of valid data were divided into perspectives of the local gaze and tourist gaze, of which 9,789 belonged to the local gaze and 10,285 to the tourist gaze. The researchers then used the ROST-CM6 software to segment the text data under the two perspectives and conducted high-frequency vocabulary analysis through keyword analysis. Finally, the top 30 words by frequency of use were selected for demonstration. Specifically, Table 1 shows the top 30 keywords based on their interactive discourse frequency within the interest-related community from the perspective of the tourist destination host. They were as follows animation world, revivifying, China, industry, resource, travel, culture, city, scene, beautiful scenery, search, art, music, guidance, animation, hotel, fine, restaurant, Tokyo, learn, inherit, brand, team, Makoto Shinkai, Suga Shrine, Gifu-ken, stunning, breakthrough, animism, and traverse. Table 2 shows the top 30 keywords based on the interactive discourse frequency of interest-related community from the perspective of tourists: tourism, Your Name, life, love, friends, animation, source, experience, photo, high score, interaction, onsite visit, anime pilgrimage, touch, shooting location, microblog, super topics, communication, fans, directors, cure, trend, position, diversify^1^, Guangdong, integration, industrial transformation, real-time, and track. The characteristics of the keywords were as follows: the high-frequency words extracted in this study are mainly nouns, and a small number of them are verbs and adjectives. Based on this, the high-frequency words in Tables 1, 2 were effectively analyzed.

**Table 1:** Interest-related community interaction based on tourism hosts (top 30).

Serial number	Keywords	Frequency	Part of speech
1	Animation world	19,870	Noun
2	Revivifying	8,115	Adjective
3	China	7,739	Noun
4	Industry	7,140	Noun
5	Resource	7,140	Noun
6	Travel	7,140	Noun
7	Culture	6,383	Noun
8	City	5,187	Noun
9	Scene	4,573	Noun
10	Beautiful scenery	4,312	Noun
11	Search	4,234	Verb
12	Art	4,234	Noun
13	Music	4,223	Noun
14	Guidance	4,003	Noun
15	Animation	3,652	Noun
16	Hotel	3,609	Noun
17	Fine	3,164	Adjective
18	Restaurant	3,080	Noun
19	Tokyo	2,958	Noun
20	Learn	2,764	Verb
21	Inherit	2,473	Verb
22	Brand	2,458	Noun
23	Team	2,347	Noun
24	Makoto Shinkai	1837	Noun
25	Suga Shrine	1786	Noun
26	Gifu-ken	1,453	Noun
27	Stunning	650	Adjective
28	Breakthrough	607	Verb
29	Animism	464	Noun
30	Traverse	431	Verb

**Table 2:** Interest-related community interaction based on tourists (top 30).

Serial number	Keywords	Frequency	Part of speech
1	Tourism	19,824	Noun
2	Your Name	19,819	Noun
3	Life	9,544	Noun
4	Love	7,933	Verb
5	Friends	7,193	Noun
6	Animation	5,154	Noun
7	Source	4,234	Noun
8	Experience	4,067	Verb
9	Photo	4,025	Noun
10	High score	3,823	Noun
11	Interaction	3,586	Noun
12	Onsite visit	3,437	Verb
13	Anime pilgrimage	3,392	Noun
14	Touch	2,984	Verb
15	Shooting location	2,905	Noun
16	Microblog	2,848	Noun
17	Super topics	2,764	Noun
18	Communication	2,764	Noun
19	Fans	2,707	Noun
20	Directors	2,676	Noun
21	Cure	2,479	Verb
22	Trend	2,206	Noun
23	Position	1875	Noun
24	Diversify	1,404	Verb
25	Mafengwo.com	1,374	Noun
26	Guangdong	790	Noun
27	Integration	778	Noun
28	Industrial transformation	640	Noun
29	Real time	607	Adjective
30	Track	454	Noun

Overall, the keywords of the interest-related community interaction from the perspective of the tourist destination hosts are mainly related to the characteristic values of the tourist destination, such as animation world, industry, resource, culture, city, beautiful scenery, art, music, hotel, restaurant, Tokyo, brand, team, Makoto Shinkai, Suga Shrine, and Gifu-ken Gifu, which indicate the existing resources and status of the tourist destination. Besides, the frequency of words such as China and travel is high, indicating that China is a huge tourism demand market; verbs such as learn and inherit imply that the tourist destination needs to explore and learn the development of film and television tourism models; and adjectives such as revivifying, fine, and stunning mainly reflect the tourists’ intuitive evaluation of the film and television works and the corresponding tourist destination.

On the other hand, the keywords of interest-related community interaction from the perspective of tourists are also mainly nouns, but the numbers of verbs and adjectives are significantly increased compared with the keywords of interest-related community interaction from the perspective of the tourist destination hosts. It is observed that the keywords from the perspective of tourists mainly focus on sharing the meaning of interactive communication, such as interaction, onsite visit, microblog, super topics, fans, friends, photo, reflecting the communication and sharing form of tourists after the tour; Moreover, the high-frequency keywords were also related to the film content, such as your name, animation, source, high score, shooting location, directors, indicating the close relationship between film and television works and tourism destinations, and reflecting that films are an effective tool to enhance the awareness of tourism destinations. Verbs such as touch and cure are suggestive of the emotional feelings that the film gives people, stimulate and drive the tourists’ attention and expectation of tourism destinations. In addition, words such as tourism, anime pilgrimage, and mafengwo.com are indicative of the tourists’ real concern about tourism destinations.

### 4.2. Element category analysis

Through the two-way analysis from the perspectives of the tourist destination hosts and tourists, and in accordance with the rules of Grounded Theory, this study first conceptualized the top 30 high-frequency words in terms of the interactive discourse frequency of interest-related community from the perspective of the tourist destination hosts and the top 30 high-frequency words in terms of the interactive discourse frequency of interest-related community from the perspective of tourists to generate the core generic categories, and the generic analysis was carried out through the three-tier coding process (open coding, relational coding, and core coding). The former was finally classified into seven secondary subcategories, which were animation attraction, cultural expression, industrial form, geographical elements, characteristics of the starting place, tourism experience and demand stimulation, and three core categories, which were tourism hotspots, tourism resources and experience demand, as shown in Table 3. The latter was classified into seven secondary subcategories, animation elements, emotional infection, life elements, environmental elements, tourism experience, destination development, and interactive communication, and three core categories, sightseeing expectation, tourism evaluation and information dissemination, as shown in Table 4.

**Table 3:** Coding of interactive elements of Interest-related community based on tourism hosts.

Level 1 coding (open login)	Level 2 coding (relational Login)	Level 3 coding (core login)
Scene, Makoto Shinkai, Animism, Traverse	Animation attraction	Tourism Hotspots
Art, Music, Animation, Learn, Inherit, Culture	Cultural expression	Tourism resources
Animation world, Industry, Resource, Restaurant, Hotel	Industrial form	
City, Tokyo, Suga Shrine, Gifu-ken	Geographical elements	
China, Travel, Search	Characteristics of the starting place	Experience demand
Beautiful scenery, Stunning, Revivifying, Fine	Tourism experience	
Team, Brand, Guidance, Breakthrough	Demand stimulation	

**Table 4:** Coding of interactive elements of Interest-related community based on tourists.

Level 1 coding (open login)	Level 2 coding (relational login)	Level 3 coding (core login)
Your Name, Animation, High score, Directors	Animation Elements	Sightseeing expectation
Touch, Cure	Emotional infection	
Life, Friends, Photo	Life Elements	
Anime pilgrimage, Shooting location, Mafengwo.com, Guangdong, Real time	Environmental elements	Tourism evaluation
Tourism, Experience, Onsite visit, Love	Tourism experience	
Trend, Position, Diversify, Integration, Industrial transformation, Track	Destination development	
Source, Interaction, Microblog, Super topics, Communication, Fans	Interactive communication	Information dissemination

Given the above analysis of high-frequency words and element categories, the development of film and television tourism should pay more attention to the construction and development of the value chain of the film and television tourism industry from the perspective of the tourist destination hosts. The film and television works are closely related to the shooting locations. When the film and television works show the unique cultural elements of the locations, they will attract social attention and extend the audiences’ demand of watching the film to field visit demand, thus stimulating a series of tourist behaviors. For example, the film *Your Name* shows the beautiful scenes of streets, stations, buildings, intersections, mountains, and lakes in Tokyo, Suga Shrine, Gifu-ken, etc., which gives the audience an immersive experience when watching the film and also stimulates the audiences’ intention to travel to the location for sightseeing and other experiences. The improvement and development of Internet platforms have also improved the interaction effect between modern film and television works and scenic spots. The online publicity of scenic spots and interactive communication of tourists have built a community of interest for the supply and demand sides. Tourism destinations should constantly improve the settings of scenic spots, improve the service capacity of the destinations, attract more tourists, and improve the economic benefits of tourism destinations. In such a way, a coupling development mode for film and television industry and tourism industry will be achieved.

From the perspective of tourists, the form of “film and television tourism” generated by the interaction between film and television works and tourism destinations can be better spread to attract tourists’ attention. As a new form of tourism, film and television tourism has a strong flow aggregation capacity, aiming to transform film and television audiences into destination tourists. For example, *Your Name* won the popularity of consumers after its first release in the cinema. With the further rendering of social media, the number of tourists going to the location will surge, thus forming a new phenomenon of film and television tourism in the new era. When tourists complete their tour of the tourist destination, they will communicate and share their own tour experience and cultural gains through WeChat, microblog, and other personal social media platforms. A new interest-related community will be formed, and information sharing and dissemination in the community will again spread to a wider range of interest-related communities. As a result, interest-related communities gradually come forth from the perspective of the destination tourists, thus realizing cultural enabling and benefit improvement that films can bring forth to tourism destinations.

## 5. Film interest-related community and tourism interest-related community

### 5.1. Discussion

Overall, with a focus on the topic of “film-enabling tourism,” the current study selected the Japanese animation film *Your Name* for online text analysis and Grounded Theory analysis and explored the film-enabling cultural tourism mechanism based on the interest-related community interaction. According to the research, in the new era of rapid development of the Internet, the emergence and interaction of interest-related community has exerted a positive impact on the sharing of film and television tourism experience and film-enabling tourism and the community effect has been greatly improved. Based on the Grounded Theory analysis, the current research generated the film-enabling tourism characteristics from the perspective of the tourist destination hosts and tourists, demonstrating that under different perspectives of destination hosts and tourists, the focuses of the film and television tourism are different. Specifically, from the perspective of the destination hosts, the focus is put on the driving force mechanism of tourist destination development and the construction and development of the value chain of the film and television tourism industry, namely, the stimulation of tourism hotspots, the excavation of tourism resources, the stimulation and satisfaction of experience demand; Whereas from the perspective of tourists, attention is paid to the guidance of the topic flow brought by the interaction between film and television works and tourism destinations based on the interest-related community, namely, the stimulation of viewing expectation, the guidance of tourism evaluation direction and the improvement of information dissemination effect. In view of the above two perspectives, the overall characteristics of the film-enabling tourism mechanisms are concluded.

**Figure 2:**
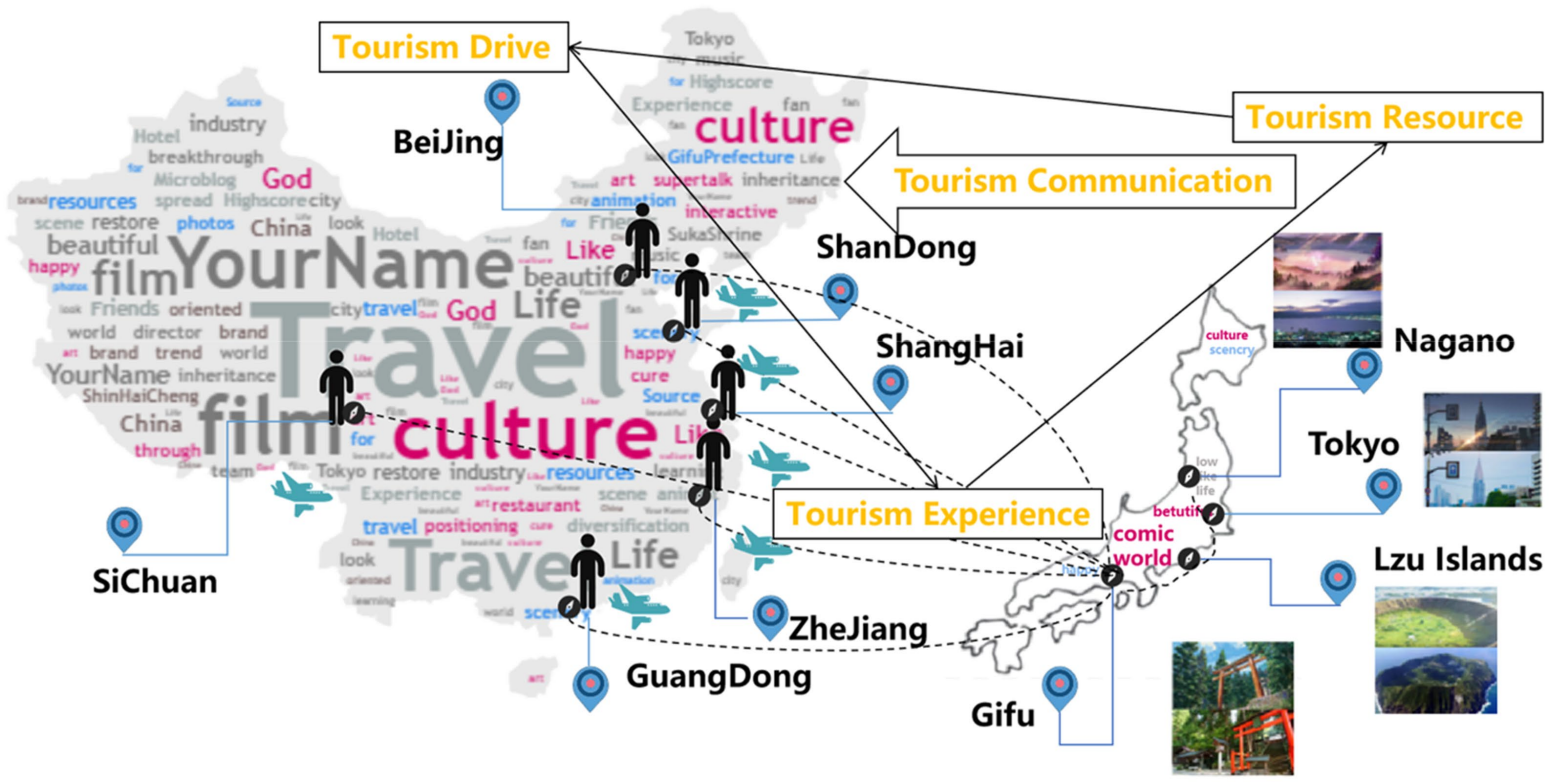
Cultural empowerment tourism of *Your Name*: based on interest-related community.

Based on the above analysis, and in consideration of the geographical location characteristics of the source of interest-related community discourse, it is revealed that most tourists are from Beijing, Shandong, Shanghai, Zhejiang, Guangdong and Sichuan, and the main tourism destinations are Nagano, Tokyo, Izu Islands and Gifu. Based on the characteristics of film-enabling tourism from the perspective of the destination hosts and tourists, this study has proposed six elements of interest-related community interaction of cultural tourism triggered by the film *Your Name*, namely tourism hotspots, tourism resources, tourist experience, sightseeing expectations, tourism evaluation and information dissemination. In such a manner, the current research has directed the pathway of cultural tourism enabled by the Japanese film *Your Name* based on the interest-related community interaction as shown in Figure 2. Films record the past, describe the present, and bear the future. The innovative development of “film and television works + tourism” is inevitable to promote the development of future cultural tourism with films. It will be a long-term sustainable development path to fully tap the value of film and television works and enable the future development of cultural tourism with film and television industry.

### 5.2. Theoretical implications

In obtaining the text data with the data crawling software, and adopting the analysis paradigm of Grounded Theory, this study has explored the film-enabling tourism mechanism based on the interest-related community interaction, analyzed the elements of the interest-related community interaction, and put forward relevant measures and suggestions. The current research has broken the limitations of previous studies based on tourism motivation and marketing perspective, innovatively introduced the concept of “interest-related community” into the research of film-enabling cultural tourism, and expanded the research perspective of film-enabling cultural tourism exploration. Previous studies discussed primarily the cultural tourism model of tourism destinations from the perspective of tourism destinations (Kim and Park, 2022, pp. 1–5; Chen, 2018, pp. 1–14) or the tourist experience (Teng, 2021, pp. 2588–2601; Teng and Chen, 2020), and rarely explored the development model of cultural tourism from the perspective of film-enabling. From the perspective of tourist destination hosts and tourists, based on the perspective of interest-related community interaction, this study has proposed six elements of interest-related community interaction, expanded the research of interest-related community in the field of film culture tourism, broadened the research perspective of interest-related community and provided a novel perspective for the future research and exploration of cultural tourism.

### 5.3. Managerial implications

This study provides important practical value for the creation of film and television works and image building of tourism destinations in the future. First of all, film-enabling cultural tourism itself is an innovative application of tourism resources, and it inspires us to strengthen the in-depth interaction between film and tourism in the future, tap into the “element resources” in film and television works, highlight the profound cultural heritage of local tourism resources in film and television works, fully use the Internet media platforms to display unique tourism culture, and create the advantages and characteristics of tourism brands, not only to attract tourists to watch films, but also to drive their strong desire to travel to the shooting locations. Moreover, it should be noted that the construction of interest-related community is the key to promoting film-enabling cultural tourism. Therefore, it is necessary to actively build interest-related community, make use of the universality of the community to further promote the film-enabling effect to cultural tourism, and achieve a significant increase in the number of film and television audiences and tourists in the interaction between the film interest-related community and tourism interest-related community. Additionally, it is also inevitable to make full use of tourism destination resources and regional resources to realize the flexible allocation, combination and expansion of tourism resources combined with film elements, open up all-round interaction mechanism between film and tourism industrial chain, and provide tourists with the whole process, full time and space experience to comprehensively cater to tourists’ demand for all-round experience and achieve innovative development.

### 5.4. Limitation and future research directions

This study still has deficiencies that need further exploration and improvement. For instance, the data collected are from social media platforms in the Chinese context, and the interest-related communities formed are mainly the audiences of the film *Your Name* and the Chinese tourists traveling to Japan, however, interest-related communities formed by tourists and local residents during the tourism process are not considered. Therefore, future research can attempt to collect data texts including local residents from international social media platforms to further analyze the discourse structure of interest-related communities formed by tourists and local residents to reflect the characteristics of a more comprehensive film-enabling cultural tourism mechanism. In addition, the latest studies in marketing encourage using multiple data sources and methodologies (Li et al., 2021; Wang et al., 2022; Zhou et al., 2023), therefore, quantitative analysis and qualitative analysis can be combined in future research to further explore the influencing factors of film-enabling cultural tourism through model construction and hypothesis verification and further analyze the influence relationship between the elements to provide objective and effective suggestions for film and television works creation and tourism destination development.

## Data availability statement

The original contributions presented in the study are included in the article/supplementary material; further inquiries can be directed to the corresponding author.

## Author contributions

YL contributed to the empirical work, the writing of the first draft, and the analysis of the results and supported the total work. JZ contributed to the revision and development of the research framework. JL contributed to the overall quality and supervision of the literature organization and empirical work. Authors discussed the results and commented on the manuscript. All authors contributed to the article and approved the submitted version.

## Funding

This work is supported by the Natural Science Foundation of China, Fund No: 72064003, project name: Study on the Regional Dynamic Evolutionary Mechanisms of Intelligent Technology, Factor Substitution and Industrial Synergistic Agglomeration.

## Conflict of interest

The authors declare that the research was conducted in the absence of any commercial or financial relationships that could be construed as a potential conflict of interest.

## Publisher’s note

All claims expressed in this article are solely those of the authors and do not necessarily represent those of their affiliated organizations, or those of the publisher, the editors and the reviewers. Any product that may be evaluated in this article, or claim that may be made by its manufacturer, is not guaranteed or endorsed by the publisher.

## Footnotes

^1^mafengwo.com
